# Oral mucositis as a pathway for fatal outcome among critically ill patients exposed to chlorhexidine: post hoc analysis of a randomized clinical trial

**DOI:** 10.1186/s13054-019-2664-6

**Published:** 2019-11-27

**Authors:** Wanessa Teixeira Bellissimo-Rodrigues, Mayra Gonçalves Menegueti, Leandro Dorigan de Macedo, Anibal Basile-Filho, Roberto Martinez, Fernando Bellissimo-Rodrigues

**Affiliations:** 1 Social Medicine Department, Ribeirão Preto Medical School, University of São Paulo, Campus Universitário, s/n, Monte Alegre, Ribeirão Preto, São Paulo 14048-900 Brazil; 2 Infection Control Service, University Hospital of Ribeirão Preto Medical School, University of São Paulo, Ribeirão Preto, Brazil; 3 Dental Service, University Hospital of Ribeirão Preto Medical School, University of São Paulo, Ribeirão Preto, Brazil; 4 Intensive Care Medicine Division, Department of Surgery and Anatomy, Ribeirão Preto Medical School, University of São Paulo, Ribeirão Preto, Brazil; 5 Infectious Diseases Division, Internal Medicine Department, Ribeirão Preto Medical School, University of São Paulo, Ribeirão Preto, Brazil

**Keywords:** Chlorhexidine, Mucositis, Mortality, Respiratory tract infections, Dental care, Critical care

Chlorhexidine (CHX) oral application has been widely used for preventing respiratory infections among critically ill patients, despite controversial effectiveness and the suspicion that it could enhance their mortality [1–3]. The physiopathology behind this association is poorly understood [2, 3]. Our objective was to reassess data from a clinical trial searching for potential pathways for the CHX-associated mortality [4, 5].

This is a post hoc analysis of a randomized clinical trial evaluating a dental care intervention aimed to prevent respiratory infections in the intensive care unit (ICU) setting. Adult patients admitted to the study ICU between January 1, 2011, and August 8, 2013, were eligible if they had a perspective of staying for 2 days. Participants were randomized by the dentist using a dice. The experimental group received dental care provided by a dentist plus routine oral care, while the control group had access only to routine oral care provided by the nursing staff. Both groups used 0.12% CHX oral solution, if fully conscious, or 2% CHX oral gel, if unconscious, three times a day throughout their ICU stay.

Adverse events potentially related to oral care procedures were pragmatically assessed at least three times a week in both study groups by the dentist during ICU stay. Their relationship with death in the ICU was evaluated through a logistic regression model, adjusting the outcome for sex, age, and Acute Physiology and Chronic Health Evaluation System II (APACHE II) score. Sample size was calculated based on the primary study outcome and the lower respiratory tract infection incidence and pointed to the inclusion of 294 patients.

Study “per protocol” population consisted of 254 patients and 9.84% (25/254) of them had adverse events related to oral care procedures, being the most common CHX-induced mucositis (7.09%, 18/254), consisting of oral erosive or ulcerative lesions, along with white plaque formation [6]. Only one patient had previous lesions before exposure to CHX. This adverse event was exclusively reported in patients exposed to 2% CHX oral gel (9.28%, 18/194, *p* = 0.006) and was found to be associated with a fatal outcome in both univariate and multivariate analysis, as shown in Table 1.

**Table 1:** Clinical and demographic characteristics of patients and occurrence of adverse events related to oral care procedures evaluated as potential risk factors for death during ICU stay

Clinical and demographic characteristics	Discharged alive	Death in the ICU	Crude RR (95%CI)	Adjusted OR (95%CI)	Adjusted *p* value
Female	69.4 (84/121)	30.6 (37/121)	–	–	–
Male	69.9 (93/133)	30.1 (40/133)	0.98 (0.68–1.43)	0.83 (0.45–1.52)	0.553
Age in years	57 (41–71)	62 (52–71)	1.02 (1.00–1.03)	1.00 (0.98–1.02)	0.781
APACHE II score	20 (16–26)	27 (21–31)	1.10 (1.06–1.15)	1.10 (1.05–1.15)	< 0.001
Routine oral care	68.5 (87/127)	31.5 (40/127)	–	–	–
Dental treatment	70.9 (90/127)	29.1 (37/127)	0.92 (0.64–1.34)	0.92 (0.50–1.67)	0.779
Without adverse events related to oral care	73.4 (168/229)	26.6 (61/229)	–	–	–
With any adverse event related to oral care	36.0 (9/25)	64.0 (16/25)	2.40 (1.67–3.46)	5.46 (2.11–14.13)	< 0.001
Without CHX-induced mucositis	72.5 (171/236)	27.5 (65/236)	–	–	–
With CHX-induced mucositis	33.3 (6/18)	66.7 (12/18)	2.42 (1.64–3.56)	6.14 (1.98–19.08)	0.002
Without intraoral bleeding	71 (174/245)	29 (71/245)	–	–	–
With intraoral bleeding	33.3 (3/9)	66.7 (6/9)	2.30 (1.40–3.80)	3.74 (0.75–18.58)	0.106

Values expressed are % (n/N) of patients for categorical variables and median (interquartile range) for continuous variables

*ICU* intensive care unit, *APACHE II* Acute Physiology and Chronic Health Evaluation System II, *RR* relative risk, *OR* odds ratio

Most of the patients who died had infection and sepsis as their direct cause of death (56/77, 72.7%). Table 2 describes the occurrence of CHX-induced mucositis and its association with direct causes of death and temporal outcomes reported during ICU stay.

**Table 2:** Occurrence of CHX-induced mucositis and its association with direct causes of death and temporal outcomes reported during ICU stay

Outcome	Without mucositis % (n/N)	With CHX-induced mucositis % (n/N)	RR (95%CI)
Death, in general	27.5 (65/236)	66.7 (12/18)	2.42 (1.64–3.56)
Death due to any infection	20.3 (48/236)	44.4 (8/18)	2.18 (1.23–3.88)
Death due to respiratory infection	10.6 (25/236)	22.2 (4/18)	2.10 (0.82–5.37)
Death due to intrabdominal infection	5.93 (14/236)	16.7 (3/18)	2.81 (0.89–8.88)
Death due to acute respiratory failure	3.4 (8/236)	5.6 (1/18)	1.64 (0.22–12.39)
Death due to cardiovascular events	2.5 (6/236)	0 (0/18)	0
Temporal outcome	Without CHX-induced mucositis Median (interquartile range)	With CHX-induced mucositis Median (interquartile range)	*p* value^a^
Duration of mechanical ventilation (days)	7 (3–16)	13 (8–20)	0.023
Duration of antimicrobial therapy (days)	5 (2–11)	12.5 (8–18)	0.002
Length of stay in the ICU (days)	7 (4–15)	14 (9–20)	0.003

*ICU* intensive care unit, *RR* relative risk

^a^Wilcoxon (Mann-Whitney test)

In the present study, we could not assess whether CHX application enhanced or not the mortality of the studied patients because all of them were exposed to it. However, examining the adverse events potentially related to oral care procedures, we found the CHX-induced mucositis was strongly and independently associated with death, even when the association was adjusted for sex, age, and the patients’ baseline severity of illness score. Consistently, patients affected by CHX-induced mucositis had a prolonged length of stay in the ICU and mechanical ventilation and were submitted to longer periods of antimicrobial therapy. Of great concern is the fact that the interruption of the 2% CHX oral gel application after identification of mucositis did not prevent these patients to clinically deteriorate, eventually leading 2/3 (12/18) of them to die in the ICU.

In conclusion, our data points to oral mucositis as the main pathway for the association between CHX exposure and enhanced in-hospital mortality. The disruption of the oral mucosa integrity possibly leads to the translocation of bacteria from the oral cavity to the bloodstream, therefore enhancing the likelihood of infection and sepsis. In our opinion, the use of oral CHX among hospitalized patients should be strictly restricted to those with established intraoral infections, such as periodontal disease, preferentially applied by a dentist.

## Data Availability

The datasets used and/or analyzed during the current study are available from the corresponding author on reasonable request.

## References

[1] Klompas M, Speck K, Howell MD, Greene LR, Berenholtz SM (2014). Reappraisal of routine oral care with chlorhexidine gluconate for patients receiving mechanical ventilation: systematic review and meta-analysis. JAMA Intern Med.

[2] Price R, MacLennan G, Glen J, SuDDICU Collaboration (2014). Selective digestive or oropharyngeal decontamination and topical oropharyngeal chlorhexidine for prevention of death in general intensive care: systematic review and network meta-analysis. BMJ.

[3] Deschepper M, Waegeman W, Eeckloo K, Vogelaers D, Blot S (2018). Effects of chlorhexidine gluconate oral care on hospital mortality: a hospital-wide, observational cohort study. Intensive Care Med.

[4] Bellissimo-Rodrigues WT, Menegueti MG, Gaspar GG, Nicolini EA, Auxiliadora-Martins M, Basile-Filho A (2014). Effectiveness of a dental care intervention in the prevention of lower respiratory tract nosocomial infections among intensive care patients: a randomized clinical trial. Infect Control Hosp Epidemiol.

[5] Bellissimo-Rodrigues WT, Menegueti MG, Gaspar GG, de Souza HCC, Auxiliadora-Martins M, Basile-Filho A (2018). Is it necessary to have a dentist within an intensive care unit team? Report of a randomised clinical trial. Int Dent J.

[6] Plantinga NL, Wittekamp BHJ, Leleu K, Depuydt P, Van den Abeele A-M, Brun-Buisson C (2016). Oral mucosal adverse events with chlorhexidine 2% mouthwash in ICU. Intensive Care Med.

